# Recent transition of medical cost and relapse rate of multiple sclerosis in Japan based on analysis of a health insurance claims database

**DOI:** 10.1186/s12883-019-1534-9

**Published:** 2019-12-16

**Authors:** Izumi Kawachi, Shuichi Okamoto, Mariko Sakamoto, Hiroyuki Ohta, Yusuke Nakamura, Kosuke Iwasaki, Manami Yoshida, Shinzo Hiroi, Mieko Ogino

**Affiliations:** 1 Department of Neurology, Brain Research Institute, Niigata University, 1-757 Asahimachi, Chuo-ku, Niigata, 951-8585 Japan; 2 Japan Medical Affairs, Takeda Pharmaceutical Company Limited, 2 Chome-1-1 Nihonbashi-Honcho, Chuo-ku, Tokyo, 103-8668 Japan; 3 Milliman, 1-6-2-8F Kojimachi, Chiyoda-ku, Tokyo, 102-0083 Japan; 4 International University of Health and Welfare, School of Medicine, Center for Medical Education, 4-2 Kozunomori, Narita, Chiba, 286-8686 Japan

**Keywords:** Multiple sclerosis, Disease-modifying therapy, Claims database, Health economics, Relapse rate, Real-world data

## Abstract

**Background:**

In this study, we aimed to understand the trends in total and itemized medical expenses, especially of disease-modifying therapy (DMT), for multiple sclerosis (MS) in Japan through an analysis of health insurance claims data.

**Methods:**

We analyzed a database containing health insurance claims data from hospitals that have adopted the Diagnosis Procedure Combination/Per-Diem Payment System in Japan. According to an algorithm based on diagnosis codes, data for all patients diagnosed with MS from April 2008 to July 2016 were extracted. Medical costs, rate of each medical treatment, and rate of relapses were analyzed by calendar-year. Medical costs in the month of relapse were compared with average medical costs per month of all MS patients by a cross-sectional analysis.

**Results:**

Four thousand three hundred seventy-four MS patients were identified in the database. Total medical cost per patient per month (PPPM) increased from ¥87,640 (US$787.7 or €723.0 as of May 2017) to ¥102,846 (US$924.4 or €848.4) during the study period. This increment was mainly attributed to the growth in cost of outpatient DMT prescriptions, which increased from ¥23,039 (US$207.1 or €190.1) to ¥51,351 (US$461.5 or €423.6). In contrast, the rate of hospitalizations and relapses PPPM decreased during the study period (from 0.053 to 0.030, and 0.032 to 0.019, respectively). Medical costs in the month of relapse (¥424,661, US$3816.8 or €3503.1) were 3.57 times higher than the average monthly costs for all MS patients (¥119,021, US$1069.8 or €981.8), with the majority comprising hospitalization cost.

**Conclusion:**

Concomitant with the increased usage of DMT, the total medical cost for treating MS is increasing in Japan. However, rates of relapse and hospitalization have shown a decreasing trend. Although this study does not show the direct causality between DMT and reduction of relapse rates/fewer hospitalizations among MS patients, a reduction in hospital costs has been revealed concomitantly with the increasing prevalence of DMT.

## Background

Multiple sclerosis (MS) is a chronic, inflammatory, demyelinating disease of the central nervous system that often affects young adults and causes progressive physical and cognitive disability [1, 2] . MS is one of the most common neurodegenerative diseases in the United States and Europe and has affected approximately 2.3 million people worldwide (in 2013) [3]. In recent years, disease-modifying therapy (DMT) has been commonly used to prevent relapse in a certain type of MS—relapsing–remitting multiple sclerosis [4, 5]. After the launch of interferon (IFN) β, the first DMT drug, in 1993, there has been rapid growth in the number of DMT drugs on the market [6]. In the United States, 12 DMT drugs are currently available. The cost of DMT has almost doubled from 2008 to 2012 in the United States, with a 5 to 7 times higher rate of annual increase compared to that of other prescription drugs [7]. The average cost of DMT per patient per month (PPPM) in 2013 was approximately $2400, comprising more than 60% of the total medical cost for MS [8]. Therefore, the cost of DMT imposes significant economic burden on healthcare systems in the United States [9].

In Asian countries, including Japan, the prevalence of MS is lower than in the United States and Europe. Therefore, MS has not received much attention from an economic perspective in Asian countries. However, the prevalence of MS has been reported to be steadily increasing in Japan and other Asian counties [10, 11]. Moreover, DMT has gained wider acceptance in recent years, leading to a steep growth in the economic impact of MS in these countries. In our previous study based on a Japanese claims database, the average medical cost PPPM for MS was ¥93,542, which was approximately 3.7-fold higher than that for the general population in Japan [12]. Furthermore, we found that DMT comprised the largest component of medical costs for patients younger than 50 years. This result demonstrated the high cost associated with MS in Japan, most of which is attributable to DMT. Given that both MS prevalence and use of DMT are increasing in Japan and other Asian countries, it is important to obtain recent information pertaining to trends in the medical costs of MS, particularly with a focus on DMT.

In this study, we conducted a calendar-year analysis to reveal the latest trends of medical cost for MS in Japan, with a special focus on DMT. We used the same type of claims database as that used in our previous study [12] and conducted a long-term analysis from 2008 to 2016 to evaluate the dynamic transition of total and itemized medical costs, which was not covered in the previous study [12]. Furthermore, we assessed the transition of the relapse rate and changes in the rates of each medical treatment and compared medical costs during the month of a relapse with the average monthly medical costs to quantify the cost of medical treatment associated with relapse. Considering the rapid growth of the DMT market, our study aims to provide useful information for healthcare providers and policymakers in choosing treatments for MS, and to conduct appropriate economic evaluations of DMT.

## Methods

### Data sources

Health insurance claims data for April 2008 to July 2016 provided by Medical Data Vision (MDV) were used in this study. The database collects data from acute care hospitals adopting the Diagnosis Procedure Combination/Per-Diem Payment System (DPC/PDPS), also known as DPC hospitals. DPC hospitals comprised approximately 21% of all hospitals and accounted for nearly 55% of total hospital beds in Japan as of May 2015. The MDV database covered approximately 11.7 million patients treated in 208 DPC hospitals all over Japan, accounting for approximately 13% of all DPC hospitals in Japan as of September 2015. As DPC hospitals are usually large hospitals, these data may be biased without the inclusion of data from small-scale hospitals and clinics. However, we reported that 67% of patients with MS in Japan visit relatively large hospitals with more than 100 beds [12]. We therefore consider the data sample from DPC hospitals in the present study to be representative of most patients with MS in Japan.

The database contains diagnosis codes according to International Classification of Disease tenth revision (ICD-10), Japanese procedure codes, and prescriptions containing generic drug names submitted for health insurance claims.

### Patient identification

**Fig. 1:**
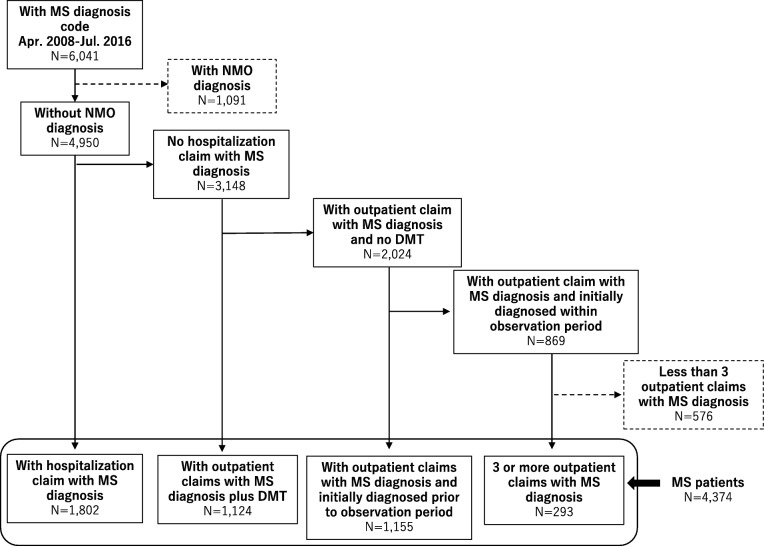
Impact of Study Inclusion/Exclusion Criteria

Patients were determined to have MS according to the ICD-10 diagnosis code G35: multiple sclerosis. Patients with MS were identified (Fig. 1) using an algorithm that has been validated in a previous study of an MS database [12], as follows:

1. Patients with at least one claim with MS diagnosis code were extracted.
2. Patients who had at least one claim with neuromyelitis optica (NMO; 3410003 by Japanese disease name codes) were excluded.
3. Patients who met any of the following criteria were included in the study population:
  * Those who had at least one hospitalization claim with MS diagnosis code.
  * Those who had at least one outpatient claim with MS diagnosis code and had at least one DMT claim.
  * Those who had at least one outpatient claim with MS diagnosis code and a record of diagnostic start date before the observation period. The diagnostic start date is defined as the day of the outpatient claim with the first diagnosis of the disease.
  * Those who had at least three outpatient claims with MS diagnosis code.

### DMT drugs

Drugs prescribed to prevent relapse of MS were defined as DMT. Eight DMT drugs were analyzed in this study, including IFN-β (including 1a, as an intramuscular injection only, and 1b), natalizumab, cyclophosphamide hydrate, azathioprine, tacrolimus hydrate, fingolimod, glatiramer acetate, and prednisolone. Cyclophosphamide hydrate, azathioprine, and tacrolimus hydrate may not be classified generally as DMT drugs; however, they are used to prevent relapse of MS in Japan. Prednisolone is used not only as a DMT drug but also to treat relapse symptoms. Therefore, prednisolone used in relapse treatment, as described in subsequent sections, was excluded from the analysis. The duration of treatment for injectable DMTs was calculated based on the days supplied per dose and the number of doses, because the number of days for injectable drug dose was not in the database. The days supplied per dose for IFN-β-1a, IFN-β-1b, natalizumab, cyclophosphamide hydrate, and glatiramer acetate were defined as 7, 2, 28, 28, and 1, respectively.

### Statistical analysis

We conducted cross-sectional analyses to examine medical costs, rate of medical treatments, and rates of relapse in each year from 2008 to 2016. The total medical cost was subdivided into inpatient and outpatient costs, and was further itemized into categories, such as medical procedure, imaging, laboratory test, DMT, prescription drugs for relapse treatment, and prescription drugs other than relapse treatment costs. Drugs used for relapse treatment were defined as injectable forms of prednisolone sodium succinate, dexamethasone phosphate ester sodium, and methylpredonisolone sodium succinate. Since data for 2016 only covers claims from January to July, we made the following adjustment: an adjustment coefficient was calculated by dividing the total medical cost in 2015 by the medical cost from January to July in 2015. This coefficient was multiplied times the medical cost from January to July for 2016 to calculate the adjusted annual cost for 2016. For calculating medical costs and rates of each medical treatment and relapse PPPM, we defined the observation period for each patient as the length of time from the first claim date with definitive MS diagnosis (either inpatient or outpatient), which is defined as the index date, to the last claim data in the dataset. The date of relapse was defined as the date of the first injection of a series of drugs used in relapse treatment at least 30 days after the index date. If multiple claims of relapse treatment were found within 30 days, only the first claim was counted as a relapse. If the interval between two relapse treatments exceeded 30 days, they were counted as separate relapses. DMT-treated patients were defined who at least once prescribed DMT in each calendar year. For these patients, medication possession ratio of DMT was calculated by dividing the number of days for which patients were supplied with DMT by the total number of days in observation period in each calendar year. Data from two different years were compared using Student’s t-tests and Chi-square tests, which were performed using SAS version 9.2 (SAS institute, Cary, NC, USA).

### Exchange rate

Following exchange rates as of May 2017 were used to convert Japanese yen to US dollar and euro described in the text; US$1 = ¥111.26, €1 = ¥121.22.

## Results

### Patient background extracted from the database

**Fig. 2:**
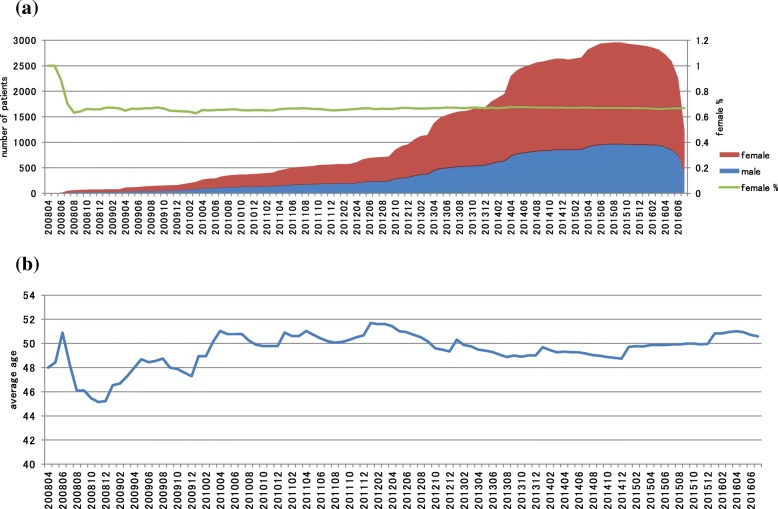
Monthly patient counts and female ratio (**a**) and average age of MS patients (**b**) by calendar year and month in the MDV database. The number of MS patients has increased in recent years and the female ratio was constant (65–67%) after August 2008 (**a**). The average age of patients was stabilized (48–52 years) after April 2010 (**b**)

We extracted 4374 patients with MS who met our study inclusion criteria (Fig. 1). Figure 2a shows the monthly patient count by gender and the female ratio of MS patients in the study population. The number of MS patients has abruptly increased in recent years because of the expansion of the database. The proportion of females was nearly constant between 65 and 67% after August 2008. The average age of patients stabilized between 48 and 52 years after April 2010 (Fig. 2b).

We counted the number of MS patients who had at least one claim for a DMT prescription in each calendar year (Table 1). The percentage of DMT-treated patients was 33% in 2008 and rapidly increased after 2012 to reach 46% in 2014; thereafter, it remained nearly stable.

**Table 1:** Number of patients prescribed DMT drugs

	2008	2009	2010	2011	2012	2013	2014	2015	2016
Number of MS patients	76	174	396	587	1000	1798	2844	3285	3459
Number of patients prescribed DMT	25	62	142	202	346	723	1314	1433	1535
% patients prescribed DMT	33%	36%	36%	34%	35%	40%	46%	44%	44%

### Medical costs by year

**Fig. 3:**
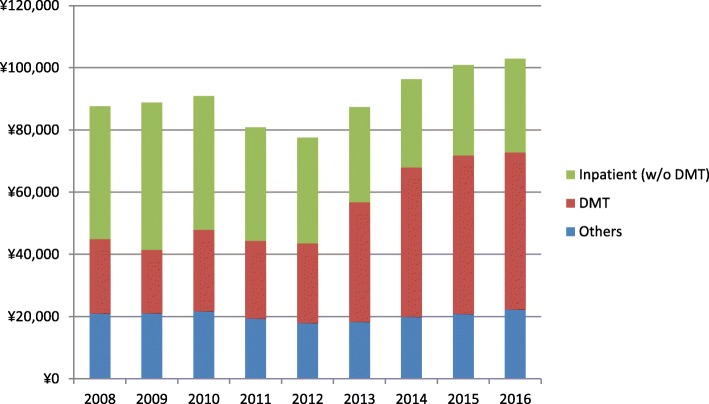
Medical costs PPPM for inpatient (without DMT), DMT, and other outpatient costs by calendar year. The percentage of DMT cost increased from 27 to 51% (*P* < 0.001), and that of inpatient cost decreased from 49 to 29% (*P* < 0.001) when comparing 2008 with 2016. DMT, disease-modifying therapy; PPPM, per patient per month

Table 2 shows total and itemized medical costs PPPM for each calendar year from 2008 to 2016. In 2008, total medical cost was ¥87,640 (US$787.7 or €723.0), and it slightly decreased to ¥77,539 (US$696.9 or €639.6) in 2012; however, it markedly increased thereafter to ¥102,846 (US$924.4 or €848.4) in 2016. During the study period from 2008 to 2016, the total outpatient cost increased from ¥44,059 (US$396.0 or €363.4) to ¥71,854 (US$645.8 or €592.7), whereas inpatient cost decreased from ¥43,581 (US$391.7 or €359.5) to ¥30,993 (US$278.6 or €255.7). Focusing our analysis on itemized expenses, we observed that the outpatient DMT cost rapidly increased from 2013 onward; it nearly doubled during the study period, from ¥23,039 (US$207.1 or €190.1) in 2008 to ¥51,351 (US$461.5 or €423.6) in 2016. Cost of outpatient imaging, laboratory tests, and prescription drugs other than DMT or relapse treatment increased marginally but not as markedly as outpatient DMT costs. Figure 3 summarizes the annual costs of inpatient (without DMT), DMT, and other medical costs. The percentage of DMT in total medical cost was 27% in 2008 but increased to 51% in 2016 (*P* < 0.001). The ratio of inpatient cost decreased from 49 to 29% (*P* < 0.001), and other medical costs did not significantly change (*P* = 0.64).

**Table 2:** Calendar-year analysis of classified medical costs PPPM for MS patients (values in 2016 were adjusted)

PPPM	2008	2009	2010	2011	2012	2013	2014	2015	2016
Total	¥87,640	¥88,839	¥90,931	¥80,813	¥77,539	¥87,358	¥96,332	¥100,937	¥102,846
Inpatient									
Total	¥43,581	¥48,008	¥43,561	¥37,393	¥34,983	¥31,847	¥29,508	¥30,235	¥30,993
Medical procedure	¥31,606	¥35,608	¥35,326	¥30,854	¥27,042	¥24,896	¥22,092	¥22,920	¥24,037
Imaging (CT, MRI)	¥1310	¥1107	¥918	¥916	¥829	¥804	¥739	¥663	¥661
Labo test	¥3315	¥2726	¥1804	¥1704	¥2060	¥1723	¥1655	¥1612	¥1712
Prescription drug (DMT)	¥901	¥597	¥529	¥937	¥987	¥1227	¥1089	¥1160	¥946
Prescription drug (relapse treatment)	¥350	¥531	¥278	¥320	¥270	¥249	¥198	¥142	¥132
Prescription drug (others)	¥6098	¥7437	¥4708	¥2662	¥3795	¥2948	¥3735	¥3739	¥3505
Outpatient									
Total	¥44,059	¥40,831	¥47,370	¥43,420	¥42,556	¥55,512	¥66,824	¥70,701	¥71,854
Medical procedure	¥5756	¥5608	¥7434	¥6620	¥5718	¥4999	¥4668	¥4134	¥4322
Imaging (CT, MRI)	¥1369	¥1423	¥1319	¥1232	¥1483	¥1944	¥1837	¥1695	¥1606
Labo test	¥1754	¥2420	¥2116	¥2217	¥2252	¥2543	¥2608	¥2633	¥2591
Prescription drug (DMT)	¥23,039	¥19,792	¥25,630	¥24,050	¥24,593	¥37,210	¥47,353	¥50,866	¥51,351
Prescription drug (relapse treatment)	¥190	¥134	¥214	¥224	¥108	¥115	¥106	¥86	¥58
Prescription drug (others)	¥11,951	¥11,454	¥10,657	¥9077	¥8402	¥8701	¥10,252	¥11,288	¥11,926
Trend of total medical cost (ratio to 2008)	1.00	1.01	1.04	0.92	0.88	1.00	1.10	1.15	1.17
Trend of inpatient cost (ratio to 2008)	1.00	1.10	1.00	0.86	0.80	0.73	0.68	0.69	0.71
Trend of outpatient DMT cost (ratio to 2008)	1.00	0.86	1.11	1.04	1.07	1.62	2.06	2.21	2.23

### Medical treatment and relapse rates by year

**Fig. 4:**
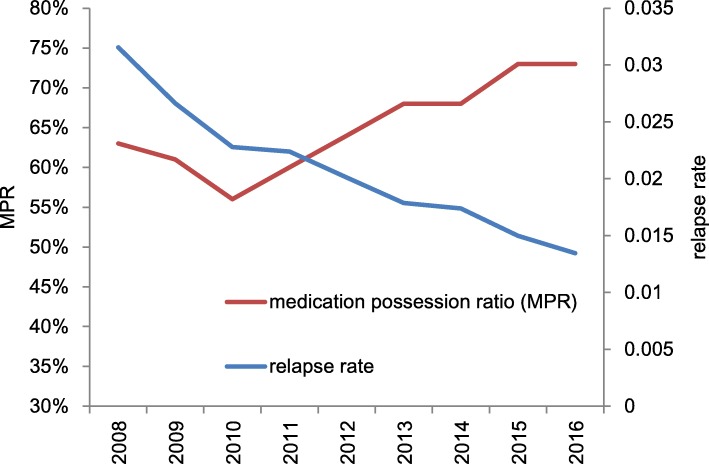
Trends of medication possession ratio of DMT and relapse rate by calendar year. The medication possession ratio of DMT increased after 2011 and relapse rate decreased during the study period. DMT, disease-modifying therapy

We next analyzed the rate (number PPPM) of each medical treatment and relapse for each calendar year from 2008 to 2016 (Table 3). The relapse rate decreased from 0.032 to 0.019 PPPM during the study period. Concomitantly, the rates of hospitalization and relapse treatment decreased from 0.053 to 0.030 PPPM and from 0.44 to 0.15 PPPM, respectively. With regard to DMT drugs, the prescription rate did not considerably change (from 0.39 to 0.35 PPPM), but days supplied per prescription increased from 18.07 to 28.64. Concomitantly, percentage of duration for which DMT-treated patients were supplied with DMT (defined as medication possession ratio) increased from 63 to 73% of the observation period (Fig. 4). Other utilization rates, including outpatient treatment, imaging, laboratory tests, and prescription drugs other than relapse treatment or DMT tended to slightly decrease; however, the changes were not as drastic as the decrease in relapse or hospitalization rates (Table 3).

**Table 3:** Usage rates of medical treatment categories and relapse PPPM (values in 2016 were adjusted)

PPPM			2008	2009	2010	2011	2012	2013	2014	2015	2016
Inpatient			0.053	0.049	0.042	0.036	0.037	0.036	0.032	0.032	0.030
Outpatient			1.06	1.07	1.08	0.99	0.95	0.90	0.91	0.86	0.83
Imaging			0.17	0.17	0.14	0.13	0.14	0.16	0.15	0.13	0.13
Lab test			0.75	0.90	0.71	0.68	0.71	0.68	0.64	0.65	0.62
Prescription drug	Relapse treatment	Number of prescription	0.44	0.46	0.33	0.32	0.26	0.17	0.17	0.15	0.15
	DMT	Number of prescription	0.39	0.33	0.40	0.39	0.34	0.39	0.40	0.36	0.35
		Days supplied/Prescription	18.07	20.82	16.06	16.13	19.46	21.58	23.56	28.31	28.64
		total days supplied	7.06	6.84	6.45	6.22	6.55	8.34	9.46	10.12	9.98
	Others	Number of prescription	7.74	8.20	7.91	7.20	6.45	5.88	5.75	5.73	5.65
Relapse			0.032	0.036	0.031	0.031	0.025	0.024	0.023	0.020	0.019

### Medical costs for patients in the month of relapse

We compared the medical costs PPPM in the month of relapse with the average medical costs PPPM for all MS patients (Table 4). In the month of relapse, total medical cost PPPM was ¥424,661 (US$3816.8 or €3503.1), which was 3.57 times higher than the cost for all MS patients (¥119,021, US$1069.8 or €981.8). Approximately 80% (¥335,556, US$3016.0 or €2768.0) of medical costs in the relapse month was inpatient cost, whereas inpatient cost comprised only 30% (¥34,877, US$313.5 or €287.7) of total MS-related costs for all MS patients. With regard to outpatient costs, expenditure on imaging and pharmacotherapy in relapse months were higher than in the average month (¥6869, US$61.7 or €56.7 vs. ¥1941, US$17.4 or €16.0; ¥2692, US$24.2 or €22.2 vs. ¥75, US$0.67 or €0.62, respectively); however, the cost of DMT was lower than the average cost per month for all MS patients (¥51,874, US$466.2 or €427.9 vs. ¥60,323, US$542.1 or €497.6).

**Table 4:** Medical costs PPPM for all MS patients and those in the month of relapse

PPPM (2016)	all MS patients	Patients with relapse
Total	¥119,021	¥424,661
Inpatient		
Total	¥34,877	¥335,556
Medical procedure	¥26,804	¥250,429
Imaging (CT, MRI)	¥792	¥11,765
Lab test	¥2028	¥21,417
Prescription drug (DMT)	¥1166	¥10,509
Prescription drug (relapse treatment)	¥162	¥4507
Prescription drug (others)	¥3924	¥36,928
Outpatient		
Total	¥84,144	¥89,105
Medical procedure	¥5112	¥9472
Imaging (CT, MRI)	¥1941	¥6869
Lab test	¥3053	¥5701
Prescription drug (DMT)	¥60,323	¥51,874
Prescription drug (relapse treatment)	¥75	¥2692
Prescription drug (others)	¥13,641	¥12,498

## Discussion

In this study, we analyzed real-world patient claims data and showed the latest trends of medical costs, relapse rates, and rates of each category of medical treatment for MS in Japan. The MDV database is one of the largest claims databases in Japan, and it enabled efficient analysis of the nationwide trends in the treatment of MS. We extracted data for 4374 MS patients from the dataset, and this is a much larger study population than that included in our previous study [12]. In 2014, the study population MS patients cover approximately 15% of the total number of MS patients in Japan [13]. The total medical cost for MS increased rapidly from 2012 to 2016, mainly due to increases in DMT cost. Concomitantly, the relapse rate as well as rate of hospitalization almost constantly decreased during the study period. Although our data did not reveal a direct causality between DMT and reduction of the relapse rate, it is demonstrated that disease progression in MS has decelerated, with longer time in remission correlated with the prevalence of DMT use.

### Increase of medical costs for MS in Japan

Our previous analysis showed that the medical cost PPPM for MS patients from October 2013 to September 2014 was ¥93,542 (US$840.8 or €771.6), of which ¥45,284 (US$407.0 or €373.6) was outpatient DMT cost [12]. This study updated these data and showed the latest trends for categories of medical costs.

The total medical cost for MS did not dramatically change from 2008 to 2012, but increased rapidly thereafter. The total increment in cost during the study period was approximately ¥15,200 (US$137 or €125) PPPM, corresponding to 17% of medical cost in 2008 (Table 2). Further, outpatient DMT cost sharply increased after 2012 and was more than twice as high by 2016. Since other medical costs did not show such drastic changes, the increment in total medical cost was mostly attributed to the growth in outpatient DMT cost. In addition, the percentage of MS patients treated with DMT rapidly increased after 2012 (35% in 2012 to 44% in 2016; Table 1), suggesting that DMT is more commonly prescribed to MS patients, including those in early disease stages due to increased recognition of its benefit [14]. In addition, the first oral DMT drug, fingolimod, was launched in November 2011 in Japan. Fingolimod now sees widespread use, comprising nearly one third of the total DMT prescription in Japan [15]. The rapid increase in DMT cost from 2012 may, at least partially, be attributable to the propagation of fingolimod.

The ratio of DMT cost to total MS-related cost in Japan increased from 27 to 51% during the study period (Fig. 3). In the United States, the cost of DMT has increased substantially over the last decade and currently constitutes more than 60% of the total MS-related cost [7, 8]. More than 67% of all MS-affected patients in the United States are estimated to be treated with DMT [8]. The prescription of DMT to patients with MS is likely to increase further in Japan. In addition, the number of DMT drugs available in Japan is expected to increase; for example, dimethyl fumarate, a new oral DMT, was approved in December 2016. Therefore, the market size as well as economic impact of DMT is likely to grow further in Japan and approach the situation in the United States.

### Relapse and medical treatment rates

In Japan, the cost of DMT has markedly increased since 2012. As the unit price of each DMT did not significantly change during the study period, either or both the supply of total DMT or/and the ratio of DMT with higher price have expanded. Although the number of DMT prescriptions did not substantially change during the study period (Table 3), days supplied per prescription increased by nearly 11 days, leading to the increase of total DMT supplies (Table 3). This partly accounts for the increase in DMT cost. In Japan, newly launched drugs cannot be prescribed for more than 14 days at a time. For fingolimod, this limitation was lifted in November 2012, and long-term prescription was permitted. In addition, natalizumab, which is normally administered every 4 weeks, was launched in June 2014. As prices of fingolimod and natalizumab are higher than prices for other conventional DMT drugs, such as IFN-β, increased prescription of these new drugs may also account for the increase of DMT cost without any change in the prescription frequency.

Concurrent with the increase of DMT cost and usage, the rate of relapses per month declined by approximately 40% (from 0.032 to 0.019) during the study period (Table 3, Fig. 4). Together with the decreased relapse rate, rates of hospitalization and relapse treatment also decreased. Although our data do not show any direct causality, the observed relationship suggests that DMT drugs reduced the incidence of relapse, which led to decreased hospitalization and relapse treatment. However, we cannot exclude the possibility that other factors, such as early diagnosis of MS, may have contributed to the reduction of the relapse rate. The DMT cost as well as supply increased sharply after 2012 (Tables 1 and 3), whereas the relapse rate constantly decreased from 2009 to 2016 (Fig. 4). This suggests that DMT alone may not have affected the relapse rate, and that other factors, such as rate of patients with mild MS symptoms, are also likely to be involved.

The reduction of inpatient cost (Table 2 and Fig. 3) is explained by the fall in relapse rate and consequent reduction of the number (or days) of hospitalizations. Despite significant decrease in inpatient cost, the increase in DMT cost exceeded this decrement, leading to a substantial increase in total MS-related medical cost.

Interestingly, we found that adherence of MS patients to DMT increased after 2011 (Fig. 4), probably due to the launch and propagation of fingolimod and natalizumab that were more convenient for patients. This may have contributed to success in treatment, leading to longer remission and less hospitalization of MS patients. Although these new drugs are costly, their utilities are recognized, and increased usage of these drugs may have inflated the recent growth of DMT cost. In the United States, expansion of DMT costs especially due to newer DMTs is a serious social problem. Although an increase in the total medical costs for MS patients is not large so far under universal health insurance in Japan, we need to continuously watch cost-effectiveness of newer DMTs awaiting clinical launch.

### Medical costs for patients with relapsing MS

Medical costs PPPM in the relapse month were 3.6-fold higher than the average medical cost PPPM for all MS patients (Table 4). This difference was mostly attributable to the difference of inpatient cost. The inpatient cost in the relapse month was approximately 10 times higher than that of the average monthly medical cost for MS. As relapse is often associated with functional impairment of patients, many patients require hospitalization for administration of acute relapse treatment. Therefore, reduction of the relapse rate not only contributes to improved quality of life for the patient but also helps reduce hospitalization costs [16].

### Limitations

Since the database included data from DPC hospitals, treatment outside the hospital were not captured. The size of the database has expanded rapidly due to the increase in DPC hospitals included in the MDV database. Therefore, the patient background may not be consistent in each calendar year; for example, the average age of the patient varied greatly before 2010 (Fig. 2b). Although clinical differences such as the severity of the disease or symptoms may affect costs, we were unable to control for these confounders as they could not be identified in the claims database. As patient data were anonymized before receipt, the history of each patient cannot be tracked either before the first visit to the hospital or after changing hospitals. For calculating costs or numbers PPPM, we defined the observation period as the length of time from the first to the last claim data for each patient and used this as the denominator. As this observation period is shorter than the insurance coverage period for each patient, the calculated value PPPM may have been an overestimate. Since only the relapses treated at the hospital that were in the database could be captured, the frequency and costs for relapses might have been underestimated. Finally, although clinical differences such as the severity of the disease or symptoms may affect costs, we were unable to control for these confounders as the information is not available in the claims database. Thus, our simply univariate analysis may not be enough to account for the underlying trend.

## Conclusions

Based on real-world data analysis, we revealed that DMT cost has grown rapidly in recent years, leading to an increase of total medical cost for MS in Japan. However, the number of relapses and hospitalizations are constantly decreasing. Although DMT is expensive, it may help reduce the relapse rate, thereby enabling lower costs due to relapsed patients, such as hospitalization and relapse treatment expenses. It is necessary to evaluate the cost of DMT in the context of total medical treatment costs, particularly considering the quality of life of MS patients [17] In Japan, an increasing number of MS patients are treated with DMT because its beneficial aspects have been increasingly recognized among both physicians and patients. The DMT market is expected to further expand in Japan as well as in other Asian countries, with a number of new DMT drugs awaiting clinical launch. Given this, our data may provide some additional information for healthcare providers and policymakers in these Asian countries for their better decision making with regard to MS treatment. In addition, the maturing process of DMT market in Japan revealed in this study from the viewpoint of both economics and utility may be a hint for other countries to evaluate and predict their own DMT market in the future.

## Data Availability

The data that support the findings of this study are available from Medical Data Vision Co., Ltd. but restrictions apply to the availability of these data, which were used under license for the current study, and so are not publicly available. Data are however available from the authors upon reasonable request and with permission of Medical Data Vision Co., Ltd.

## Abbreviations

DPC/PDPS: Diagnosis Procedure Combination/Per Diem Payment System
ICD-10: International Classification of Disease 10th edition
IFN: Interferon
MDV: Medical Data Vision
MS: Multiple sclerosis
NMO: Neuromyelitis optica
PPPM: Per patient per month
